# Clinical outcomes of neoadjuvant chemotherapy for resectable colorectal liver metastasis with intermediate risk of postoperative recurrence: A multi‐institutional retrospective study

**DOI:** 10.1002/ags3.12631

**Published:** 2022-10-21

**Authors:** Takehiro Noda, Hidekazu Takahashi, Mitsuyoshi Tei, Naohiro Nishida, Taishi Hata, Yutaka Takeda, Masayuki Ohue, Hiroshi Wada, Tsunekazu Mizushima, Tadafumi Asaoka, Mamoru Uemura, Shogo Kobayashi, Kohei Murata, Taroh Satoh, Yuichiro Doki, Hidetoshi Eguchi, the Clinical Study Group of Osaka University (CSGO), Colorectal Group, and Hepato‐Biliary‐Pancreatic Group, Masaaki Miyo, Kenji Sakai, Masanori Tujie, Yoshihito Ide, Osakuni Morimoto, Ken Nakata, Shin Nakahira, Kohtarou Kidani, Kazuhiko Hashimoto, Shinichi Yoshioka, Yasuji Hashimoto, Tomoya Kishimoto, Katsuki Danno, Keishi Sugimoto, Masakazu Ikenaga, Terumasa Yamada, Takamichi Komori, Shigekazu Yokoyama, Hiroyoshi Takemoto, Satoshi Ohshima, Hirotoshi Kim, Masahiro Tanemura, Shingo Noura, Junzo Shimizu, Yujiro Fujie, Satoshi Hyuga, Shu Okamura, Nariaki Fukuchi, Nobuo Tanaka

**Affiliations:** ^1^ Department of Gastroenterological Surgery, Graduate School of Medicine Osaka University Suita Japan; ^2^ Department of Surgery Osaka Rosai Hospital Sakai Japan; ^3^ Department of Medical Oncology Osaka International Cancer Institute Osaka Japan; ^4^ Department of Surgery Kansai Rosai Hospital Amagasaki Japan; ^5^ Department of Gastroenterological Surgery Osaka International Cancer Institute Osaka Japan; ^6^ Department of Surgery Osaka Police Hospital Osaka Japan

**Keywords:** colon cancer, colorectal live metastasis, neoadjuvant chemotherapy, propensity score, resection

## Abstract

**Aims:**

Risk‐scoring systems for colorectal liver metastasis (CRLM) after hepatectomy allow prognoses to be predicted preoperatively. We investigated the clinical outcomes of neoadjuvant chemotherapy for resectable CRLM according to patient risk status, aiming to determine the subgroup of patients who could benefit from neoadjuvant chemotherapy.

**Methods:**

In this multi‐institutional retrospective analysis, the preoperative risk score was calculated from six previously reported factors: synchronous metastases, primary lymph node positivity, tumor number, largest tumor diameter, extrahepatic metastasis, and the preoperative carbohydrate antigen 19–9 level. Patients were divided into three groups according to their risk scores: low risk (score = 0), intermediate risk (score 1–10), and high risk (score ≥11). Overall and recurrence‐free survival curves were calculated using the Kaplan–Meier method. After propensity‐score matching in the intermediate‐risk group, we compared clinicopathological features and outcomes.

**Results:**

There were 318 cases, from 20 institutions. The preoperative risk score could be calculated in 277 cases. There were 34, 192, and 51 patients in the low‐, intermediate‐, and high‐risk groups, respectively. Intermediate‐risk group patients who received neoadjuvant chemotherapy had significantly better recurrence‐free survival than that of patients without neoadjuvant chemotherapy (*P* = .0453). After propensity‐score matching in the intermediate‐risk group, the recurrence‐free survival rate was better in patients who received neoadjuvant chemotherapy (*P* = .0261). But the overall survival rate was not improved after the matching.

**Conclusion:**

Neoadjuvant chemotherapy for resectable CRLM might prolong the recurrence‐free survival period for intermediate‐risk patients with preoperative risk scores in the range of 1–10, but the overall survival was not improved by neoadjuvant chemotherapy.

## Graphical abstract

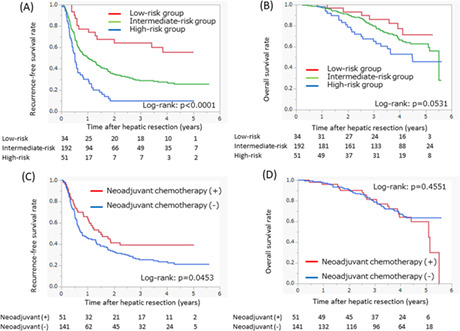

## 1 INTRODUCTION

Colorectal liver metastasis (CRLM) is a major cause of death and case numbers are increasing worldwide. Hepatic resection is the only treatment for CRLM with curative potential, and the 5‐y survival rate after hepatectomy is reported to be around 50%.^1^, ^2^ However, the recurrence rate after hepatectomy is still high, and the most frequent site of tumor relapse is the remnant liver.^3^, ^4^, ^5^

In recent years, neoadjuvant chemotherapy has been applied to several types of malignancy and has improved patients' prognosis.^6^ Neoadjuvant chemotherapy has been shown to prolong recurrence‐free survival or overall survival compared with upfront surgery by suppressing tumor micrometastasis and increasing surgical resectability.^7^, ^8^ However, neoadjuvant chemotherapy has the disadvantages of tumor progression and liver toxicity, including sinusoidal obstruction and steatosis.^9^, ^10^ For CRLM, Nordlinger et al reported that perioperative chemotherapy with 5‐fluorouracil/folinic acid and oxaliplatin (FOLFOX4) increased progression‐free survival compared with hepatic resection alone. However, the final analysis demonstrated that perioperative chemotherapy did not benefit patients' survival.^6^, ^11^ The European Society for Medical Oncology (ESMO) consensus guidelines indicate that perioperative treatment might not be necessary in cases diagnosed as resectable CRLM with favorable prognosis, but that perioperative chemotherapy should be administered to patients with resectable CRLM when the prognosis is unclear or unfavorable.^12^ Thus, the major issue is how to select patients with resectable CRLM for neoadjuvant chemotherapy or surgery alone.

Several risk‐scoring systems for CRLM after hepatectomy have been developed recently.^13^, ^14^, ^15^ Patients' prognosis after hepatectomy for CRLM can be predicted preoperatively. We therefore investigated the clinical outcomes of neoadjuvant chemotherapy for resectable CRLM according to risk status after hepatectomy in a large number of patients recruited for a multi‐institutional retrospective analysis, with the objective of determining the subgroup of patients who could benefit from neoadjuvant chemotherapy for CRLM.

## 2 MATERIALS AND METHODS

### 2.1 Patient enrollment

**FIGURE 1:**
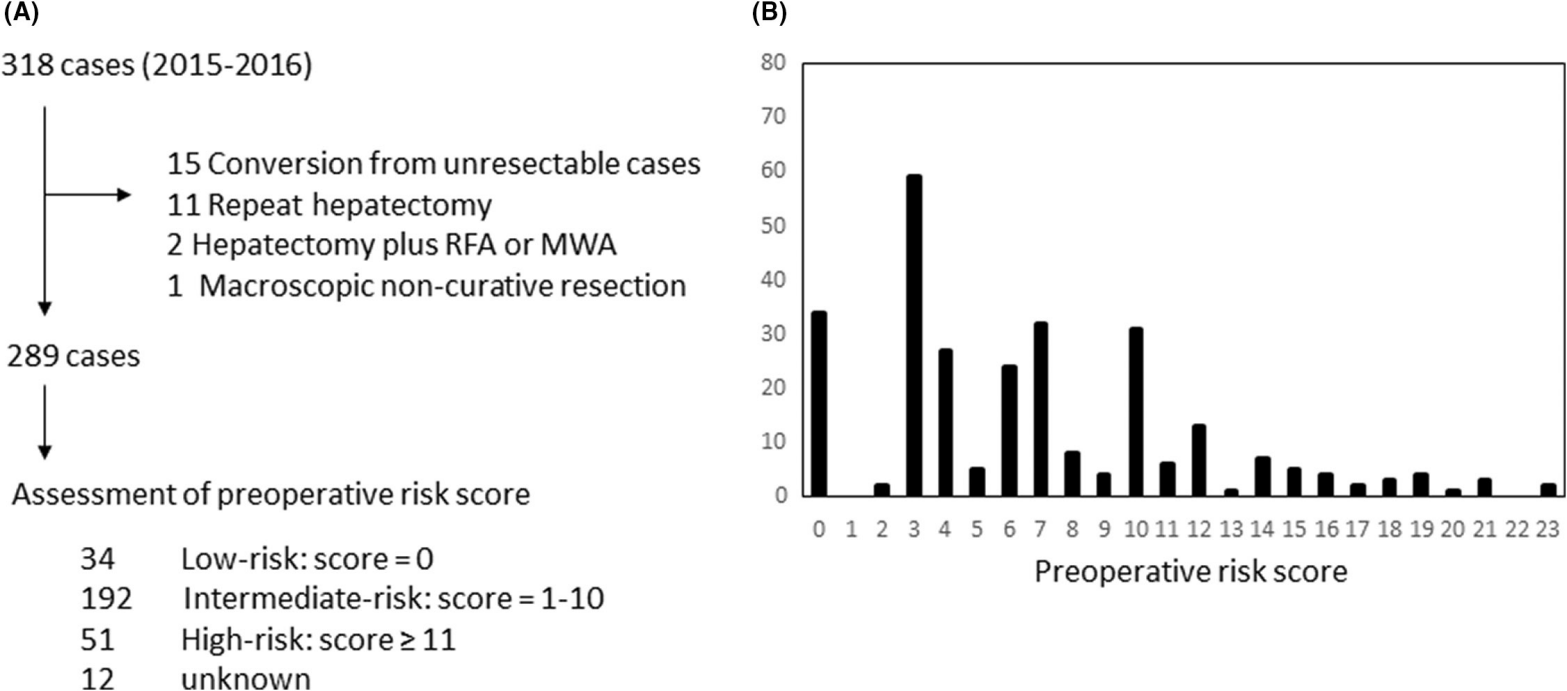
Flowchart of the study and the distribution of preoperative risk scores. (A) The schema of this study and the results of assessment of preoperative risk. (B) The distribution of preoperative risk scores

This study was a multi‐institutional retrospective analysis. The enrolled cases underwent hepatic resection for CRLM between January 2015 and December 2016 at the institutions belonging to the Clinical Study Group of Osaka University, Hepato‐Biliary‐Pancreatic Group, and Colorectal Group. In total, 318 cases were registered from 20 institutions. The surgeons of these institutes joined the seminars of surgical skills for the unification of surgical techniques. Moreover, the certified expert surgeons or supervising surgeons of the Japanese Society of Hepato‐Biliary‐Pancreatic Surgery attended the operations in most of institutes. Thus, the surgical techniques are standardized and quality of operation were secured in both high‐volume hospitals (more than 50 liver resections per year) and medium‐volume hospitals (<50 liver resections per year). Information about the characteristics and clinical courses of the patients was obtained from patient medical records at each medical institution. A flowchart of the analysis is shown in Figure 1A. From 318 cases, 29 cases including conversion cases from unresectable liver metastasis (n = 15), repeat hepatectomy (n = 11), hepatectomy plus radiofrequency ablation (RFA), or microwave ablation (MWA; n = 2) and macroscopic noncurative hepatic resection (n = 1) were excluded. Finally, 289 cases were selected for analysis. This retrospective study was approved by the Institutional Review Board of Osaka University Hospital and each institution (No: 20076). The study was conducted in accordance with the Declaration of Helsinki.

### 2.2 Data collection

The following clinicopathological data were collected: sex, age, Eastern Cooperative Oncology Group (ECOG) performance status, carcinoembryonic antigen (CEA), and carbohydrate antigen 19–9 (CA19‐9) before treatment of the metastatic hepatic lesion, primary lesion location, presence of preoperative treatment for primary lesion and regimens, maximum diameter of the primary lesion, lymph node dissection, histology of the primary lesion, pathological T factor, pathological N factor, pathological stage, R factor, curability at primary lesion resection, KRAS status, presence of adjuvant chemotherapy after resection of the primary lesion, timing of liver metastasis, timing of resection of the liver metastasis, number of liver metastases, maximum diameter of liver metastasis, distribution of liver metastasis, presence of extrahepatic metastasis, H factor, grade, presence of neoadjuvant chemotherapy for the metastatic hepatic lesion, regimens and treatment cycles, operative approach for liver metastasis, types of hepatic resection, operative times, blood loss, postoperative hospital stay, presence of complication above grade III, histological response, presence of adjuvant chemotherapy, regimens, and cycles. In the Japanese classification system of colorectal carcinoma, H factor was defined by the number of hepatic lesions and tumor size. Pathological T factor, N factor, stage, R factor, and curability were defined by the Japanese Classification of Colorectal, Appendiceal, and Anal Carcinoma. Grade was defined by H factor and the nodal status of the primary lesion.^16^ Types of hepatic resection were defined by the General Rules for the Clinical and Pathological Study of Primary Liver Cancer.^17^ Postoperative complications were graded according to the extended Clavien–Dindo classification of surgical complications published by the Japan Clinical Oncology Group and its postoperative complications criteria.^18^

### 2.3 Assessment of preoperative risk score

The preoperative risk score was calculated from six preoperative factors based on the nomogram for disease‐free survival, as previously reported.^15^ The six factors were the following: synchronous metastases (3 points), primary lymph node positive (3 points), number of tumors 2–4 (4 points) and ≥5 (9 points), largest tumor diameter over 5 cm (2 points), extrahepatic metastasis at hepatectomy (4 points), and preoperative carbohydrate antigen 19–9 level over 100 (4 points). The total points of the preoperative score ranged from 0 to 25. The patients were divided into three groups according to the preoperative risk score: a low‐risk group (score = 0), an intermediate‐risk group (score 1–10), and a high‐risk group (score ≥11).

### 2.4 Propensity‐score matching analysis

In the intermediate‐risk group, the patients receiving neoadjuvant chemotherapy, had a favorable prognosis of higher recurrence‐free survival compared with the patients without neoadjuvant chemotherapy. However, the background characteristics in the intermediate‐risk group varied in the patients receiving neoadjuvant chemotherapy or surgery alone. Thus, propensity‐score matching analysis was conducted for the intermediate‐risk group. Two factors of timing of liver metastasis and maximum tumor diameter of the metastatic lesion affected the clinical decision for performing neoadjuvant chemotherapy for the patients with CRLM and these two factors were selected for propensity‐score matching analysis. The caliber for matching was set as 0.2. There were 192 patients in the intermediate‐risk group and 51 patients treated with neoadjuvant chemotherapy and 141 patients treated with surgery alone. One‐to‐one matching is most commonly implemented, but the ratio of treated and untreated patients in this study was unbalanced to be about 1:3. Thus, we performed 1:3 matching of patients with neoadjuvant chemotherapy and without neoadjuvant chemotherapy, using optimal matching.^19^, ^20^ The standardized mean difference (SMD) was used to assess the balance of the clinical backgrounds between the two groups. SMD of <0.2 was considered an adequate balance, and SMD between 0.2 and 0.8 indicated the medium differences. SMD >0.8 meant considerable differences. Analyses were performed using the EZR software program, which is a graphical user interface for R.^21^

### 2.5 Statistical analysis

Statistical analyses were performed according to the prescribed protocol of the clinical trial. All data are expressed as mean ± standard deviation. Statistical differences between the groups were analyzed using Student's *t*‐test for continuous variables and the chi‐square test for other variables. Overall survival and disease‐free survival curves were computed using the Kaplan–Meier method, and differences between survival curves were compared using the log‐rank test. All statistical analyses were conducted using JMP Pro (v. 15.1.0; SAS Institute, Cary, NC, USA). All *P* values < .05 were considered statistically significant.

## 3 RESULTS

### 3.1 Patient characteristics

A total of 289 patients with CRLM treated by liver resection were analyzed in this study. Table 1 showed the characteristics of these patients with resectable CRLM. The patients were 168 males and 121 females. The median age was 68 y old (range 31–90). Preoperative treatment for the primary lesion was conducted in 18 patients, and the regimens were capecitabine plus oxaliplatin (XELOX, n = 10); FOLFOX (n = 6); 5‐fluorouracil/folinic acid, oxaliplatin, and irinotecan (FOLFOXIRI, n = 1); and 5‐fluorouracil/folinic acid and irinotecan (FOLFIRI, n = 1). The median maximum diameter of the primary lesion was 50 mm, and most of the patients received lymph node dissection. After resection of the primary lesion, 79 patients received adjuvant chemotherapy. There were 119 patients who had synchronous liver metastasis and 170 who had metachronous liver metastasis. The number of liver metastases was single (n = 158) or multiple (n = 131). In 28 patients, there were more than five metastatic nodules in the liver. The median maximum diameter of liver lesions was 20 mm, and the distribution of liver metastasis was unilobar (n = 209) or bilobar (n = 80). Thirty‐four patients had extrahepatic metastasis. Eighty‐four patients received neoadjuvant chemotherapy before resection of the liver metastatic lesion. The regimens were XELOX (n = 37), FOLFOX (n = 27), FOLFIRI (n = 5), FOLFOXIRI (n = 4), and others (n = 11); S‐1 plus oxaliplatin (n = 3), capecitabine (n = 2), tegafur/uracil (n = 2), S‐1 (n = 1), camptothecin (n = 1), camptothecin plus S‐1 (n = 1), and camptothecin plus capecitabine (n = 1). Molecular‐targeted drugs were added in 66 patients. The clinical responses to neoadjuvant chemotherapy were complete response (CR, n = 1), partial response (PR, n = 24), stable disease (SD, n = 29), progressive disease (PD, n = 22), and not assessed (NA, n = 8). The types of hepatic resection were nonanatomical resection (Hr0, n = 174), sectionectomy (Hrs, n = 24), segmentectomy (Hr1, n = 55), two segmentectomy (Hr2, n = 35), and three segmentectomy (Hr3, n = 1). The median operative time was 288 min, and the median volume of blood loss was 250 mL. The median postoperative hospital stay was 11 d. Postoperative complication above Grade III was observed in 18 cases. Adjuvant chemotherapy was conducted in 75 cases. The regimens were XELOX (n = 36), FOLFOX (n = 10), FOLFIRI (n = 3), and others (n = 26). Molecular‐targeted drugs were added in 19 patients.

**TABLE 1:** Characteristics of patients with resectable CRLM

Variables		All cases (n = 289)
Sex	Male:Female	168:121
Age (y)		68 (31–90)
PS (ECOG)	0:1:2:3	245:37:6:1
CEA (ng/mL)		8.7 (0.6–13 323)
CA19‐9 (IU/mL)		18 (0.6–13 576)
Location	Colon:Rectum	173:116
	Right side:Left side	74:215
Preoperative treatment for primary lesion	Present:Absent:unclear	18:269:2
	Regimens	XELOX 10, FOLFOX 6, FOLFOXIRI 1, FOLFIRI 1
	Molecular‐targeted drug	Bevacizumab 10, Panitumumab 2
Maximum diameter of primary lesion (mm)		50 (12–130)
Lymph node dissection	D0:D1:D2:D3:DX	1:4:23:252:6
Histology of primary lesion	tub1:tub2: others	79:191:10
pT factor	T1:T2:T3:T4a:T4b	10:12:168:70:22
pN factor	N0: N1a: N1b: N2a: N2b: N3	99:54:58:37:24:11
pStage	I:IIa: IIb: IIc: IIIa: IIIb: IIIc: IVa: IVb: IVc	12: 44: 7: 5: 3: 68: 23: 105: 11: 2
R factor	R0:R1:R2	188:3:93
Curability	CurA: CurB: CurC	159:26:100
KRAS	Wild: mutant: unknown	94: 74: 121
Adjuvant chemotherapy	Present:Absent	79:95
Timing of liver metastasis	Synchronous:Metachronous	119:170
Timing of resection of liver metastasis	Simultaneous resection:Asynchronous	24:92
Number of liver metastases	Single:Multiple	158:131
	1–4 nodules: over 5 nodules	261:28
Maximum diameter of metastatic lesion (mm)		20 (2–180)
	<50 mm:≥50 mm	254:32
Distribution of liver metastasis	Unilobar:Bilobar	209:80
Extrahepatic metastasis	Present:Absent	34:255
H factor	H1:H2:H3:HX	236:44:6:3
Grade	A:B:C	173:80:27
Neoadjuvant chemotherapy for metastatic lesion	Present:Absent	84:205
	Regimens	XELOX 37, FOLFOX 27, FOLFIRI 5, FOLFOXIRI 4, others 11
	Molecular‐targeted drug	Bevacizumab 47, Panitumumab 14, Cetuximab 5
Number of cycles		5 (1–35), mean 7.1
Response	CR: PR: SD: PD:NA	1:24:29:22:8
Operative approach for liver metastasis	OPEN: LAP: HYBRID: HALS	181: 99: 7: 2
Types of hepatic resection	Hr0:Hrs:Hr1:Hr2:Hr3	174: 24: 55: 35:1
Operative times (min)		288 (57–1166)
Blood loss (mL)		250 (0–4375)
Postoperative hospital stay (d)		11 (3–212)
Complication (>Grade III)	Present:Absent	18:271
Histological response	Grade0: 1a: 1b: 2: 3: unknown	8:12:12:13:2:23
Adjuvant chemotherapy	Present:Absent	75:214
	Regimens	XELOX 36, FOLFOX 10, FOLFIRI 3, others 26
	Molecular‐targeted drug	Bevacizumab 17, Panitumumab 2
Number of cycles		6 (1–42), mean 6.6

Abbreviations: CRLM, colorectal liver metastasis; CA19‐9, carbohydrate antigen 19–‐9; CEA, carcinoembryonic antigen; CR, complete response; FOLFIRI, 5‐fluorouracil/folinic acid and irinotecan; FOLFOX, 5‐fluorouracil/folinic acid and oxaliplatin; FOLFOXIRI, 5‐fluorouracil/folinic acid, oxaliplatin, and irinotecan; HALS, hand‐assisted laparoscopic surgery; HYBRID, hybrid hepatectomy; LAP, laparoscopic hepatectomy; NA, not assessed; OPEN, open hepatectomy; PD, progressive disease; PR, partial response; PS (ECOG), performance status (Eastern Cooperative Oncology Group); SD, stable disease; XELOX, capecitabine plus oxaliplatin.

### 3.2 Distribution of preoperative risk score

Among the 289 cases, the preoperative risk score could be calculated in 277 cases. In 12 cases, the preoperative risk score could not be calculated because of missing values. The median preoperative risk score was 6; the distribution is presented in Figure 1B. The patients were divided into three groups according to their preoperative risk scores: a low‐risk group (preoperative risk score = 0, n = 34), an intermediate‐risk group (preoperative risk score 1–10, n = 192), and a high‐risk group (preoperative risk score ≥11, n = 51). The characteristics of the groups are summarized in Table 2. The factors of sex, performance status, the location of the primary lesion, and the maximum diameter of the primary lesion did not differ significantly among the three groups. The percentages of cases with high CEA (over 10 ng/mL) and high CA19‐9 (over 100 IU/mL) were significantly higher in the high‐risk group. In the low‐risk group, all cases had metachronous and single liver metastasis without extrahepatic metastasis. In the intermediate‐risk group, 78 cases (41%) had synchronous metastasis and multiple liver metastases. The maximum diameter of the metastatic lesion was larger, at 26.3 mm. In the high‐risk group, 39 cases (76%) had synchronous metastasis and 48 cases (94%) had multiple liver metastases. The maximum diameter of the metastatic lesion was the largest, at 40.6 mm.

**TABLE 2:** Patients' characteristics according to preoperative risk score

Variables		Low‐risk group score: 0 (n = 34)	Intermediate‐risk group score: 1–10 (n = 192)	High‐risk group score: ≥11 (n = 51)	*P* value
Sex	Male:Female	25:9	112:80	27:24	.1513
Age (y)	<65:≥65	6:28	79:113	24:27	.0162
PS (ECOG)	0:1:2:3	28:4:1:1	162:27:3:0	45:4:2:0	.1404
CEA (ng/mL)	<10:≥10	25:9	102:87	15:34	.0003
CA19‐9 (IU/mL)	<100:≥100	34:0	173:14	22:26	<.0001
Location	Colon:Rectum	21:13	112:80	31:20	.9024
	Right side:Left side	6:28	50:142	14:37	.5392
Maximum diameter of primary lesion (mm)	<50:≥50	16:17	85:97	19:29	.6375
pT factor	T1:T2/3:T4	3:27:3	5:121:64	0:30:21	.0025
pN factor	N0:N+	34:0	55:137	8:43	<.0001
KRAS	Wild type: mutant: unknown	3: 7: 24	69: 41: 81	21: 20: 10	<.0001
Timing of liver metastasis	Synchronous:Metachronous	0:34	78:114	39:12	<.0001
Number of liver metastases	Single:Multiple	34:0	114:78	3:48	<.0001
	1–4 nodules: over 5 nodules	34:0	191:1	26:25	<.0001
Maximum diameter of metastatic lesion (mm)		18.7 ± 7.0	26.3 ± 21.9	40.6 ± 29.8	<.0001
	<50 mm:≥50 mm	34:0	176:16	35:16	<.0001
Extrahepatic metastasis	Present:Absent	34:0	13:179	34:17	<.0001
H factor	H1:H2:H3:HX	34:0:0:0	176:16:0:0	17:28:6:0	<.0001
Grade	A:B:C	34:0:0	125:53:14	11:27:13	<.0001
Neoadjuvant chemotherapy for metastatic lesion	Present:Absent	1:33	51:141	30:21	<.0001
Operative approach for liver metastasis	OPEN: LAP: HYBRID: HALS	16:18:0:0	114:72:5:1	41:7:2:1	.0093
Types of hepatic resection	Hr0:Hrs:Hr1:Hr2:Hr3	24:6:3:1:0	128:12:40:12:0	17:7:9:17:1	<.0001
Operative times (min)		239 ± 121	314 ± 157	367 ± 148	.0006
Blood loss (mL)		333 ± 551	420 ± 590	690 ± 825	.0140
Postoperative hospital stay (d)		18.3 ± 34.6	13.3 ± 9.5	13.8 ± 5.8	.1844
Complication (>Grade III)	Present:Absent	2:32	12:180	2:49	.8178
Adjuvant chemotherapy	Present:Absent	8:26	46:146	20:31	.0824

Abbreviations: CRLM, colorectal liver metastasis; CA19‐9, carbohydrate antigen 19–‐9; CEA, carcinoembryonic antigen; HALS, hand‐assisted laparoscopic surgery; HYBRID, hybrid hepatectomy; LAP, laparoscopic hepatectomy; OPEN, open hepatectomy; PS (ECOG), performance status (Eastern Cooperative Oncology Group).

### 3.3 Short‐term surgical outcomes

Nonanatomical hepatic resection (Hr0) was conducted in 24 cases (71%) in the low‐risk group, in 128 cases (67%) in the intermediate‐risk group, and in 17 cases (33%) in the high‐risk group. The operative times were significantly longer in the high‐risk group (239 min in the low‐risk group, 314 min in the intermediate‐risk group, and 367 min in the high‐risk group; *P* = .0006), and blood loss was larger in the high‐risk group (333 mL in the low‐risk group, 420 mL in the intermediate‐risk group, and 690 mL in the high‐risk group; *P* = .0140). However, the periods of postoperative hospital stay were not significantly different (18.3 d in the low‐risk group, 13.3 d in the intermediate‐risk group, and 13.8 d in the high‐risk group; *P* = .1844). The rates of postoperative complication above grade III were not significantly different, being 6% in the low‐risk group, 6% in the intermediate‐risk group, and 4% in the high‐risk group.

### 3.4 Long‐term surgical outcomes

**FIGURE 2:**
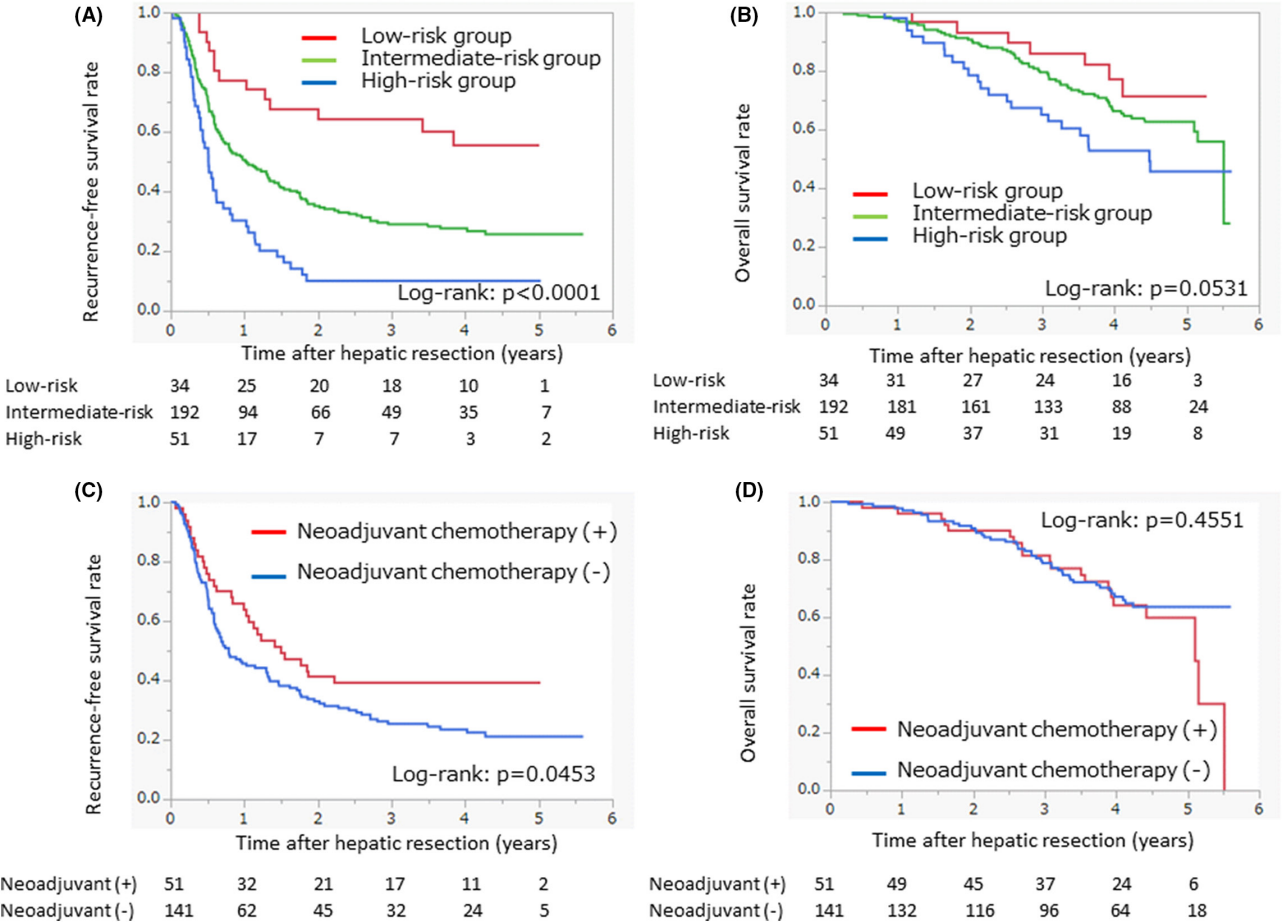
Surgical outcomes after hepatic resection. (A,B) Recurrence‐free survival curves and overall survival curves of the patients grouped by preoperative risk score. Red line: Low‐risk group (score = 0), green line: Intermediate‐risk group (score 1–10), blue line: High‐risk group (score ≥11). (C,D) Recurrence‐free survival curves and overall survival curves of the intermediate‐risk groups with and without neoadjuvant chemotherapy

The recurrence‐free survival curves and overall survival curves of the three groups are shown in Figure 2A,B. The 2‐y recurrence‐free survival rates were 68% in the low‐risk group, 35% in the intermediate‐risk group, and 10% in the high‐risk group. The 3‐y and 5‐y overall survival rates were, respectively, 86% and 71% in the low‐risk group, 80% and 63% in the intermediate‐risk group, and 65% and 46% in the high‐risk group. Before hepatic resection for the metastatic lesion(s), 84 cases received neoadjuvant chemotherapy. The percentage of cases receiving neoadjuvant chemotherapy was 59% in the high‐risk group and 27% in the intermediate‐risk group, but only 3% in the low‐risk group. The recurrence‐free survival curves and overall survival curves of each group according to the presence of neoadjuvant chemotherapy are presented in Figure 2C,D (the intermediate‐risk group), Figure S1A–D (the low‐ and high‐risk group). In the low‐risk group, the 2‐y recurrence‐free survival rates were 100% of cases receiving neoadjuvant chemotherapy and 67% of cases without neoadjuvant chemotherapy, but these rates were not significantly different (*P* = .4370). The 3‐y overall survival rates were 100% in cases receiving neoadjuvant chemotherapy and 85% in cases without neoadjuvant chemotherapy (*P* = .5563; Figure S1A,B). In the intermediate‐risk group, the patients receiving neoadjuvant chemotherapy had better recurrence‐free survival compared with the patients without neoadjuvant chemotherapy (*P* = .0453; Figure 2C). The 2‐y recurrence‐free survival rates were 41% of cases receiving neoadjuvant chemotherapy and 33% of cases without neoadjuvant chemotherapy. The 3‐y overall survival rates were 81% in cases receiving neoadjuvant chemotherapy and 79% in cases without neoadjuvant chemotherapy (*P* = .4551; Figure 2D). In the high‐risk group, the patients receiving neoadjuvant chemotherapy had a recurrence‐free survival rate similar to that of the patients without neoadjuvant chemotherapy (*P* = .9287). The patients receiving neoadjuvant chemotherapy had a 3‐y overall survival rate similar to that of the patients without neoadjuvant chemotherapy (67% vs 63%, *P* = .7440; Figure S1C,D).

In each risk group, the overall survival curves between high volume hospital and medium volume hospital aere shown (Figure S2A–C). In the low‐risk group, the 2‐y recurrence‐free survival rates were 75% in the high volume hospital and 63% in the medium volume hospital (*P* = 0.2262) and the 3‐y overall survival rates were 92% in the high volume hospital and 83% in the medium volume hospital (*P* = .1698; Figure S2A). In the intermediate‐risk group, the 2‐y recurrence‐free survival rates were 30% in the high volume hospital and 38% in the medium volume hospital (*P* = .1901) and the 3‐y overall survival rates were 82% in the high volume hospital and 78% in the medium volume hospital (*P* = .1752; Figure S2B). In the high‐risk group, the 2‐y recurrence‐free survival rates were 7.3% in the high volume hospital and 14% in the medium volume hospital (*P* = .4705) and the 3‐y overall survival rates were 71% in the high volume hospital and 58% in the medium volume hospital (*P* = .6962; Figure S2C). In all risk groups, the 2‐y recurrence‐free survival rates and the 3‐y overall survival rates were not significant between high volume and medium volume hospital.

### 3.5 Propensity‐score matching analysis

**FIGURE 3:**
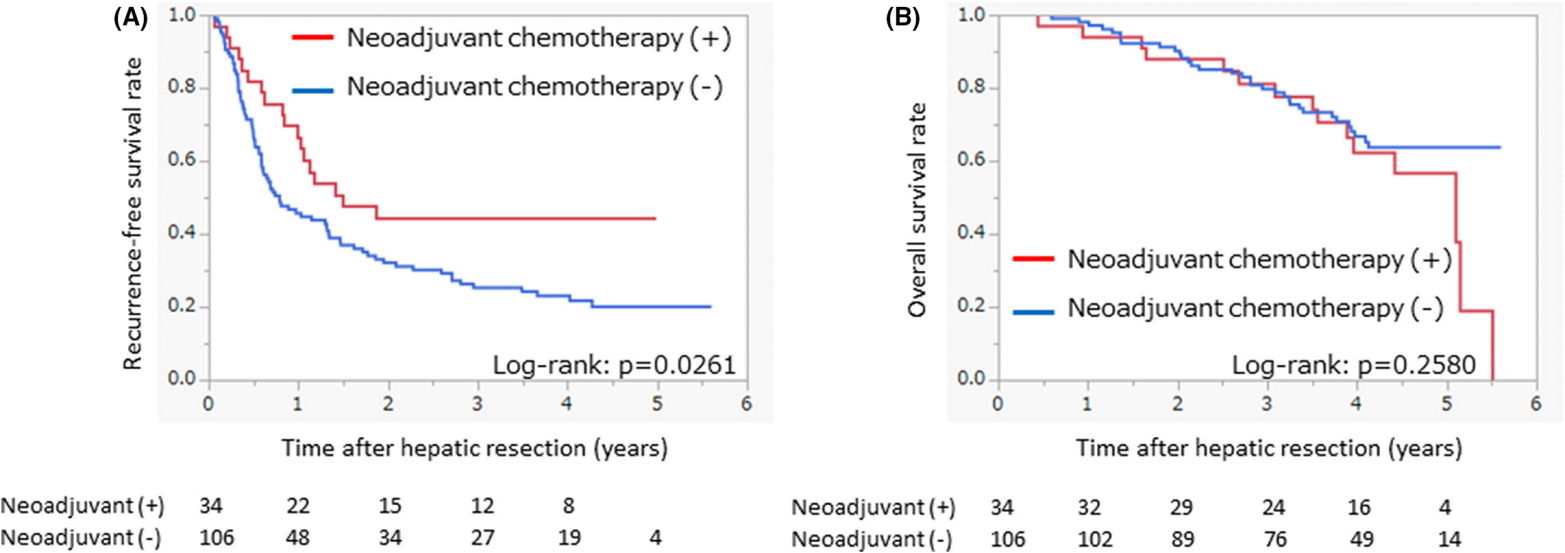
Recurrence‐free survival curves and overall survival curves of the intermediate‐risk group after propensity‐score matching. (A) Recurrence‐free survival curves of the intermediate‐risk group with and without neoadjuvant chemotherapy after propensity‐score matching. (B) Overall survival curves of the intermediate‐risk group with and without neoadjuvant chemotherapy after propensity‐score matching

In the intermediate‐risk group, the patients receiving neoadjuvant chemotherapy had a favorable prognosis of higher recurrence‐free survival compared with the patients without neoadjuvant chemotherapy. Propensity‐score matching analysis was conducted for the intermediate‐risk group; from 192 patients, 140 were selected, among whom 34 patients received neoadjuvant chemotherapy and 106 patients received surgery alone. The patients' characteristics and tumor characteristics before and after propensity‐score matching are shown in Table 3. There were no significant differences in characteristics observed between the two matched groups. The short‐term surgical outcomes of operative time, blood loss, period of postoperative hospital stay, and morbidity did not differ significantly between the two groups. The recurrence‐free survival curves and overall survival curves after propensity‐score matching are shown in Figure 3A,B. The patients who received neoadjuvant chemotherapy had better recurrence‐free survival when compared with the patients without neoadjuvant chemotherapy (*P* = .0261), and the 2‐y recurrence‐free survival rates were 44% in patients who received neoadjuvant chemotherapy and 32% in patients without neoadjuvant chemotherapy. After propensity‐score matching of the intermediate‐risk group, the 3‐y overall survival rates were 81% in patients who received neoadjuvant chemotherapy and 80% in patients without neoadjuvant chemotherapy (*P* = .2580).

**TABLE 3:** Patients' characteristics of resectable CRLM with preoperative risk score (1–10) according to neoadjuvant chemotherapy

Variables		Before PSM				After PSM			
		NAC (+) (n = 51)	NAC (−) (n = 141)	*P* value	SMD	NAC (+) (n = 34)	NAC (−) (n = 106)	*P* value	SMD
Sex	Male:Female	31:20	81:60	0.6787	.068	18:16	60:46	.7087	.074
Age (y)	<65:≥65	24:27	55:86	0.3184	.163	15:19	43:63	.7150	.072
PS (ECOG)	0:1:2	39:12:0	123:15:3	.0492	0.282	25:9:0	91:12:3	.0601	0.310
CEA (ng/mL)	<10:≥10	19:29	83:58	.0206	0.443	14:19	61:45	.1283	0.332
CA19‐9 (IU/mL)	<100:≥100	40:6	133:8	.0991	0.476	28:3	101:5	.3273	0.419
Location	Colon:Rectum	28:23	84:57	0.5619	.095	18:16	62:44	.5703	.112
	Right side:Left side	11:40	39:102	0.3957	.142	8:26	29:77	.6568	.088
Maxium diameter of primary lesion (mm)	<50:≥50	21:27	64:70	0.6327	.085	14:18	49:54	.7047	.102
pT factor	T1:T2/3:T4	1:29:20	4:92:44	0.5393	0.215	0:22:11	3:72:31	.4131	.067
pN factor	N0:N+	16:35	39:102	0.6152	.081	13:21	26:80	.1285	0.299
KRAS	wild: mutant: unknown	27: 12: 12	42: 29: 70	.0028	0.484	15: 10: 9	36: 21: 49	.1120	0.209
Timing of liver metastasis	Synchronous:Metachronous	30:21	48:93	.0020	0.513	16:18	48:58	.8565	.036
Number of liver metastasis	Single:Multiple	32:19	82:59	0.5674	.094	22:12	66:40	.7972	.051
	1–4 nodules: over 5 nodules	50:1	141:0	.0955	0.200	33:1	106:0	.0912	0.246
Maximum diameter of metastatic lesion (mm)		36.1 ± 35.9	22.8 ± 12.0	.0002	0.496	23.8 ± 14.4	23.2 ± 12.7	.8213	.043
	<50 mm:≥50 mm	42:9	134:7	.0050	0.409	32:2	100:6	.9614	.010
Extrahepatic metastasis	Present:Absent	4:47	9:132	0.7221	.057	4:30	7:99	.3505	.179
H factor	H1:H2	42:9	134:7	.0050	0.409	31:3	100:6	.5268	.122
Grade	A:B:C	29:16:6	96:37:8	0.2245	0.233	27:5:2	69:31:6	.2084	0.324
Operative approach for liver metastasis	OPEN: Lap: Hybrid: HALS	34:15:2:0	80:57:3:1	0.4474	0.205	23:10:1:0	62:40:3:1	.6994	.191
Types of hepatic resection	Hr0:Hrs:Hr1:Hr2	32:3:12:4	96:9:28:8	0.8733	.112	23:3:8:0	74:5:20:7	.1815	.047
Operative times (min)		348 ± 198	302 ± 137	.0721	0.270	309 ± 154	298 ± 135	.7063	.072
Blood loss (ml)		568 ± 746	366 ± 514	.0355	0.316	493 ± 533	388 ± 547	.3286	.195
Postoperative hospital stay (d)		13.6 ± 5.9	13.2 ± 10.5	0.7524	.058	14.1 ± 6.0	13.3 ± 11.7	.6941	.089
Complication(over Grade III)	Present:Absent	3:48	9:132	0.8993	.021	3:31	8:98	.812	.047
Adjuvant chemotherpy	Present:Absent	9:42	37:104	0.2179	0.209	4:30	30:76	.0385	0.422

Abbreviations: CRLM, colorectal liver metastasis; CA19‐9, carbohydrate antigen 19–9; CEA, carcinoembryonic antigen; HALS, hand‐assisted laparoscopic surgery; HYBRID, hybrid hepatectomy; LAP, laparoscopic hepatectomy; NAC, neoadjuvant chemotherapy; OPEN, open hepatectomy; PS (ECOG), performance status (Eastern Cooperative Oncology Group); PSM, propensity‐score matching; SMD, standardized mean difference.

## 4 DISCUSSION

This multi‐institutional retrospective study demonstrated that patients with resectable colorectal liver metastasis with intermediate risk of postoperative recurrence had a significantly better recurrence‐free survival if they received neoadjuvant chemotherapy, as shown by propensity‐score matching analysis. However, overall survival was not improved by neoadjuvant chemotherapy in patients with intermediate risk. Meanwhile, in patients with resectable colorectal liver metastasis and either a low or high risk of recurrence, neoadjuvant chemotherapy did not improve recurrence‐free survival or overall survival.

Regarding the effect of neoadjuvant chemotherapy in patients with resectable colorectal liver metastasis, several studies have been reported.^22^, ^23^ In 2013, the EORTC intergroup trial 40 983 showed that perioperative chemotherapy with FOLFOX4 increased progression‐free survival compared with surgery alone in patients with resectable liver metastases from colorectal cancer, but had no overall survival benefit.^6^, ^11^ The subsequent exploratory retrospective analysis found that the baseline factors of highly elevated CEA value, performance status, and lower body mass index predicted a benefit of perioperative chemotherapy.^24^ Some recent large‐scale studies have used clinically applicable scoring systems in reporting extensive experience of liver resection for metastatic colorectal cancer. Fong et al identified seven factors as significant and independent predictors of poor long‐term outcome, including positive margin, extrahepatic disease, node‐positive primary tumor, disease‐free interval from primary to metastases, number of hepatic tumors, largest hepatic tumor, and CEA level. They established a scoring system for dividing the patients into a group with favorable outcomes of hepatic resection and a group considered for experimental adjuvant or neoadjuvant trials.^14^ Beppu et al identified six preoperative factors associated with overall survival (synchronous metastases, positive primary lymph node, number of tumors, largest tumor diameter, extrahepatic metastasis at hepatectomy, and preoperative CA19‐9 level) and established a nomogram to determine the likelihood of early recurrence and the necessity for perioperative chemotherapy.^15^ The nomogram is very simple and convenient for assessment of the risk of tumor recurrence. However, it was established from clinical data between 2000 and 2004. The recent advance of chemotherapy regimens might influence the interpretation of the risk status of the nomogram. Ono et al conducted the retrospective analysis for resectable colorectal liver metastasis and reported that Beppu et als nomogram score over 6 was an independent risk factor for tumor recurrence and it could be a good tool for predicting the prognosis of patients with CRLM after the improvement of chemotherapeutic regimens.^25^ We assessed the preoperative score from Beppu et al's nomogram and divided our cohort into three ranked groups having low risk, intermediate risk, and high risk. In the low‐risk group, all patients had a metachronous solitary metastatic tumor less than 5 cm in diameter, and only one case received neoadjuvant chemotherapy. This group was supposed to have a favorable prognosis, and upfront surgery without neoadjuvant chemotherapy might have been desirable.

In the intermediate‐risk group, the patients with neoadjuvant chemotherapy showed significantly longer recurrence‐free survival compared with the patients without neoadjuvant chemotherapy.However, no benefit to overall survival from neoadjuvant chemotherapy was observed. We have considered the several reasons that longer recurrence‐free survival did not lead to improvement of overall survival. First, that study was a retrospective observational multi‐institutional study by collecting the clinical data of the patients for 2 y, 2015–2016. But the regimens of neoadjuvant chemotherapy and the treatment duration varied in each institution. In actuality, 51 patients received neoadjuvant chemotherapy and the regimens were mainly XELOX (47%) and FOLFOX (25%). The fixed protocol of neoadjuvant chemotherapy for the intermediate‐risk group of CRLM patients had the possibility to show the benefit to overall survival. Second, recent advances in chemotherapy, molecular‐targeted drugs, surgical managements, and genomic analysis for metastatic colorectal cancer might have contributed to the patients' survival, since tumor recurrence after hepatic resection.^3^, ^26^, ^27^ Even if the patients had unresectable tumor relapse after the resection of CRLM, many chemotherapeutic regimens and combination therapies with molecular‐targeted drugs were available. Some patients with unresectable diseases could show an excellent response and receive the conversion surgery.^28^, ^29^ These curative treatments for recurrent lesions or surgical treatment for other recurrent lesions, such pulmonary metastasis or lymph node metastasis, might have contributed to the overall survival. In this study, the date for the treatments administered for the recurrent lesions were not obtained. Further study will need the clinical data of the second or third treatments for the recurrent lesion after hepatic resection.

To select patients for treatment by neoadjuvant chemotherapy for CRLM, several authors have stratified them by preoperative risk status or by several markers that were available before hepatectomy^30^, ^32^, ^33^. Ayez et al reported that CRLM patients with high clinical risk scores, as established by Fong^14^, benefited from neoadjuvant chemotherapy and that patients with low risk profiles might not benefit from neoadjuvant chemotherapy.^30^ Based on these results, the authors planned a prospective randomized clinical trial to evaluate the impact of neoadjuvant chemotherapy in high‐risk patients with primary resectable CRLM, and that trial is now in progress.^31^ Matsumura et al conducted a multicenter, randomized, phase III trial to compare surgery followed by a postoperative FOLFOX regimen with surgery followed by a perioperative FOLFOX regimen plus cetuximab in patients with KRAS wildtype resectable CRLM. KRAS status was used to stratify patients for perioperative chemotherapy, but there was no significant difference in progression‐free survival or overall survival between the perioperative chemotherapy plus cetuximab group and the postoperative chemotherapy group.^32^ Ninomiya et al focused on the CRLM grading system that is currently endorsed by the Japanese Society for Cancer of the Colon and Rectum. This grading system involves not only information about the hepatic tumor but also the nodal status of the primary lesion. They defined patients with synchronous and grade B/C CRLM as a high‐risk subgroup and demonstrated that their prognosis could benefit from neoadjuvant chemotherapy in comparison with upfront surgery.^33^ These grades B and C are based on the number of hepatic lesions, their maximum diameter, and the nodal status of the primary tumor. The preoperative risk score reported by Beppu et al^15^ was calculated from six factors (synchronous metastases, primary lymph node positivity, number of hepatic tumors, largest tumor diameter, extrahepatic metastasis at hepatectomy, and CA19‐9 level) and included the grade B/C factors listed above. In our study, we found that the patients in the intermediate‐risk group could be stratified as good candidates for neoadjuvant chemotherapy and that neoadjuvant chemotherapy might prolong their recurrence‐free survival. For the patients in the low‐risk group, our results demonstrate that upfront surgery without neoadjuvant chemotherapy might be desirable. A prospective randomized controlled trial is needed for verification.

In conclusion, we found that neoadjuvant chemotherapy for initially resectable CRLM might prolong the recurrence‐free survival period for patients in the intermediate‐risk group, with preoperative risk scores in the range of 1–10. However, the overall survival was not improved by neoadjuvant chemotherapy in patients with intermediate risk.

## DISCLOSURE

Funding: This research received no specific funding.

Conflict of Interest: Takehiro Noda, Hidekazu Takahashi, Mitsuyoshi Tei, Naohiro Nishida, Kohei Murata, Yutaka Takeda, Masayuki Ohue, Hiroshi Wada, Tadafumi Asaoka, Mamoru Uemura, Shogo Kobayashi, Taro Sato, Yuichiro Doki, and Hidetoshi Eguchi have no conflicts of interest or financial ties to disclose. Tsunekazu Mizushima has conflicts of interest or financial ties to disclose. Tsunekazu Mizushima and Yuichiro Doki are editorial board members of AGS.

Author Contributions: TN, HT, MT, and NN conceived, devised and designed the study. TH, YT, MO, HW, TM, TA, MU, SK, KM, TA, YD, and HE contributed to data collection and interpretation and critically reviewed the article. All authors approved the final article.

Ethics Statements (Humans Ethics Approval Declaration): This retrospective study was approved by the Institutional Review Board of Osaka University Hospital and each institution (No: 20076). The study was conducted in accordance with the Declaration of Helsinki and good clinical practice.

## Supporting information


Figure S1

Figure S2
