# Supplementary figures and images for: Clinical outcomes of neoadjuvant chemotherapy for resectable colorectal liver metastasis with intermediate risk of postoperative recurrence: A multi‐institutional retrospective study
Source: Ann Gastroenterol Surg. 2022 Oct 21;7(3):479–90. doi: 10.1002/ags3.12631 (PMC10154835; doi:10.1002/ags3.12631)

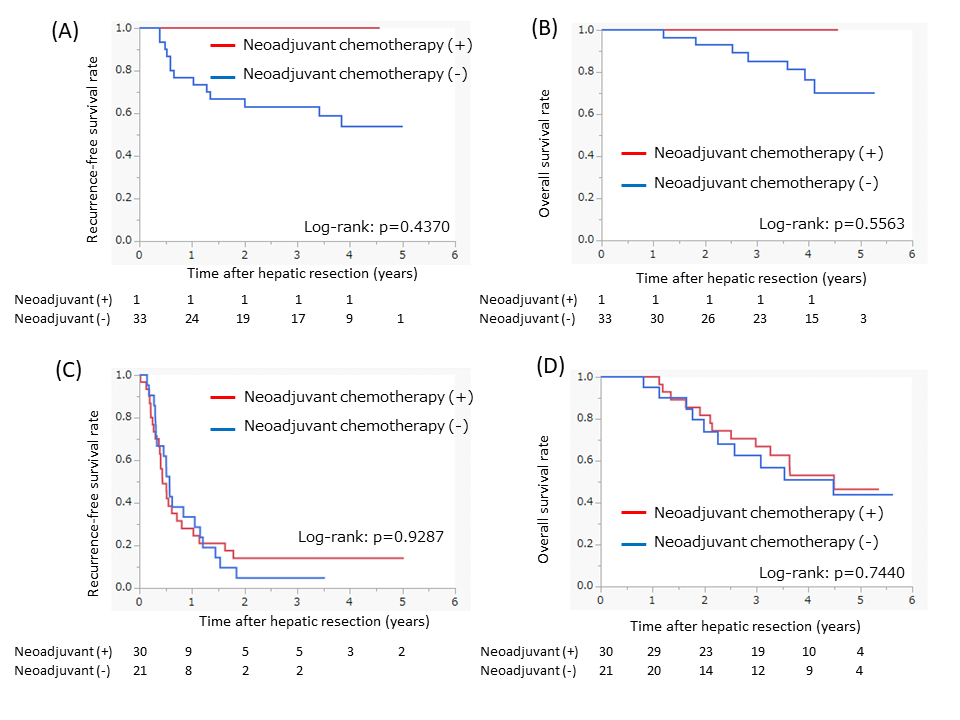

Supplement: Supplementary file 1 — Figure S1 [file AGS3-7-479-s001.TIF]

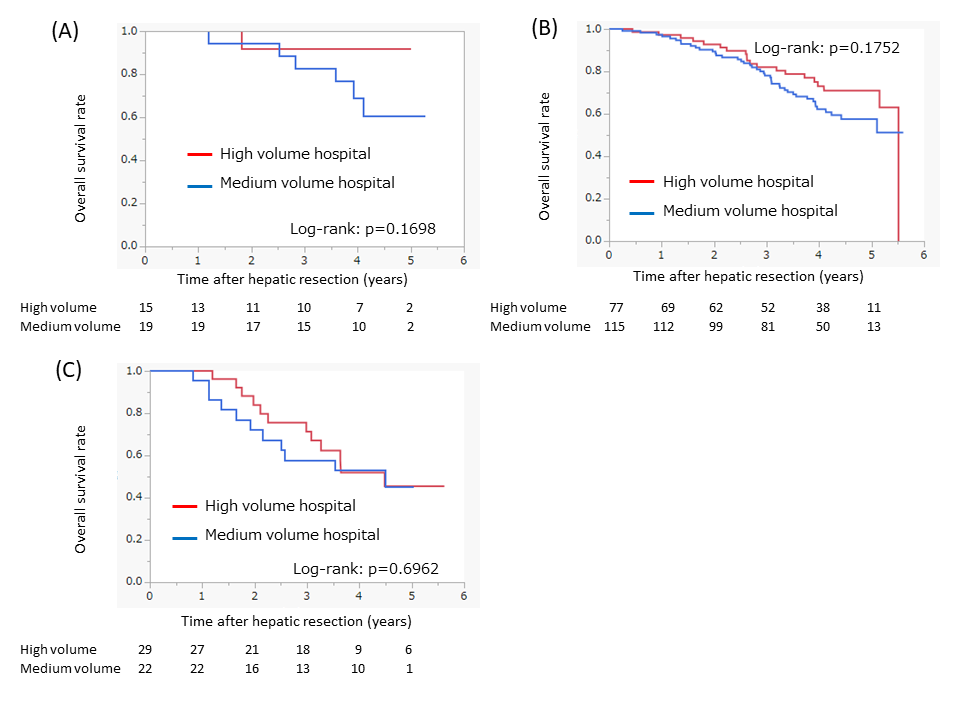

Supplement: Supplementary file 2 — Figure S2 [file AGS3-7-479-s002.TIF]
